# Various modes of action of dietary phytochemicals, sulforaphane and phenethyl isothiocyanate, on pathogenic bacteria

**DOI:** 10.1038/s41598-019-50216-x

**Published:** 2019-09-23

**Authors:** Dariusz Nowicki, Monika Maciąg-Dorszyńska, Krystyna Bogucka, Agnieszka Szalewska-Pałasz, Anna Herman-Antosiewicz

**Affiliations:** 1 Department of Bacterial Molecular Genetics, Faculty of Biology, University of Gdańsk, Wita Stwosza 59, 80-308 Gdańsk, Poland; 2 Department of Medical Biology and Genetics, Faculty of Biology, University of Gdańsk, Wita Stwosza 59, 80-308 Gdańsk, Poland

**Keywords:** Antimicrobials, Pathogens

## Abstract

Isothiocyanates (ITCs) derived from cruciferous plants reveal antibacterial activity, although detailed mechanism is not fully elucidated. Recently it has been reported that ITCs induce the stringent response in *Escherichia coli* strains. The aim of this work was to determine whether two isothiocyanates, sulforaphane (SFN) and phenethyl isothiocyanate (PEITC), similarly as in *E*. *coli* induce stringent response in *Bacillus subtilis*, model Gram(+) bacterium, and test their potency against a panel of clinical isolates belonging to Gram(+) or Gram(−) groups. Minimal inhibitory concentrations were determined as well as effect of ITCs on membranes integrity, synthesis of DNA, RNA and stringent response alarmones was assessed. SFN and PEITC are effective against *B*. *subtilis* and bacterial isolates, namely *E*. *coli*, *K*. *pneumonia*, *S*. *aureus*, *S*. *epidermidis* and *E*. *faecalis*. Interestingly, in *B*. *subtilis* and *E*. *faecalis* the inhibition of growth and nucleic acids synthesis is independent of ppGpp accumulation. In bacteria, which do not induce the stringent response in the presence of ITCs, membrane integrity disruption is observed. Thus, ITCs are effective against different pathogenic bacteria and act by at least two mechanisms depending on bacteria species.

## Introduction

Over the last few decades infections caused by bacteria resistant to commonly used antibiotics became one of the major health care problems. At the same time, efforts to develop new antibacterial drugs by pharmaceutical industries have declined substantially. Thus, an increasing number of patients suffer from diseases caused by bacteria which are not sensitive to currently available drugs. It results in increased morbidity and mortality, longer hospitalization and health care costs^1^.

Plants pose a valuable source of new biologically active compounds with antibacterial activity. Among them, isothiocyanates (ITCs) have received increasing attention due to their chemopreventive, anticancer as well as antimicrobial activities coupled with moderate toxicity towards normal human cells^2,3^. They are generated from glucosinolates, secondary metabolites found abundantly in plants from *Brassicaceae* family, which are hydrolyzed by a β-thioglucosidase (myrosinase) released after plant tissue damage, or myrosinase derived from mammalian gastrointestinal tract microflora^4^.

One of the first investigated ITCs was allyl isothiocyanate (AITC) which has been shown to effectively inhibit different pathogenic microorganisms^5^. Its use was approved in Japan for food preservation^6^. Another aliphatic ITC, sulforaphane (SFN), has shown activity against *Helicobacter pylori* both *in vitro* and *in vivo*^7^. It also effectively inhibited a panel of Gram(−) bacteria, including *Escherichia coli* and *Pseudomonas aeruginosa*, and Gram(+) bacteria, such as *Enterococcus faecalis*, *Staphylococcus aureus* or *S*. *saprophyticus* isolated from human gastrointestinal tract^8^. Antimicrobial activity of aromatic ITC, 2-phenylethyl isothiocyanate (PEITC), has been described for harmful intestinal bacteria (*Clostridium difficile*, *Cl*. *perfringens*, *E*. *coli*) while it had no effect on growth of beneficial bifidobacteria and lactobacilli, as assessed using disc-diffusion assay^9^. In another study, agar disc diffusion assay showed that PEITC was more effective against Gram(+) bacteria (*Bacillus cereus*, *B*. *subtilis*, *Listeria monocytogenes*. *S*. *aureus*) than Gram(−) bacteria (*Aeromonas hydrophila*, *P*. *aeruginosa*, *Salmonella choleraesuis*, *S*. *enterica*, *Serratia marcescens*, *Shigella sonnei*, *Vibrio parahaemolyticus*)^10^. Recently, ten ITCs naturally found in cruciferous vegetables, including SFN, AITC, BITC and PEITC, have been tested for 14 bacterial strains growing in BHI medium (*B*. *cereus*, *B*. *subtilis*, *E*. *faecalis*, *E faecium*, *Lactobacillus plantarum*, *L*. *monocytogenes*, *S*. *aureus*, *S*. *xylosus*). Results indicated that in most cases bacteria were sensitive to the ITC tested, however the extent of antimicrobial activity depended on the ITC and the strain considered^11^.

Most reports are limited to determination of minimal inhibitory concentration (MIC), which may vary depending on a method used. There is no general rule concerning the efficacy of ITCs toward various bacteria but aromatic ITCs seem to be more active that aliphatic ones^11^. The mechanism of antimicrobial action of ITCs is not well understood. ITC group is highly electrophilic and reacts with amine, thiol or hydroxyl groups, thus it may bind to cellular targets influencing their activity and function. For instance, AITC inhibited activity of thioredoxin reductase and acetate kinase, which are responsible for important metabolic reactions in bacteria^12^, however, existence of ITC-enzyme conjugates has not been proven. It has been also reported that phenyl ITC (PITC), AITC and PEITC had strong antimicrobial activity through the disruption of the bacterial cell membrane function which led to a loss of its integrity and subsequent cell death^13,14^. Recently, it has been shown that PEITC, SFN, AITC, benzyl isothiocyanate (BITC), PITC and isopropyl isothiocyanate (IPRITC) efficiently inhibited growth of Shiga toxin harboring *E*. *coli* strains which was accompanied by an inhibition of the lytic development of Shiga toxin-converting bacteriophage and *stx* gene expression. Moreover, these effects were mediated by alarmones of the stringent response^15,16^.

The stringent response is one of the most far-reaching global regulatory mechanisms in bacteria. The unusual nucleotides, ppGpp and pppGpp (referred together to as (p)ppGpp) are synthesized promptly after the onset of starvation and various physical and chemical stresses. In addition to the stress response, (p)ppGpp regulates also other processes such as biofilm formation, quorum sensing, and bacterial virulence^17,18^. The stringent response is aimed to reprogram cellular metabolism in order to downregulate the active growth and energy consuming processes and to activate those necessary for survival and adaptation to the challenging environmental conditions. For instance, during amino acid starvation, transcription from promoters of genes coding for stable RNA (rRNA or tRNA) or ribosomal proteins is inhibited and transcription from promoters for amino acids transport and biosynthesis is activated^17,19^. One of crucial processes which are affected under conditions of the stringent response is DNA replication. Its downregulation, however, may be achieved at initiation or elongation stage, thus it might not be detectable shortly after stringent response induction but after longer times^20^.

(p)ppGpp metabolism in the cell is under the control of specific enzymes able to synthesize and hydrolyze these nucleotides. *E*. *coli* and some of other gamma- and beta-proteobacteria harbor two enzymes, synthetase RelA (specialized in response to amino acid starvation) and bifunctional SpoT, responsible for both synthesis and hydrolysis of (p)ppGpp. While most of other bacterial species, including Gram(+) bacteria, have single bifunctional enzyme, named generally RSH (Rel/Spo homolog)^21^. Nevertheless, in both genetic arrangements, dysfunction of these enzymes is not lethal for bacteria, but leads to the distinct phenotype, named ppGpp-null, characterized by impaired stress response, decreased survival in nutrient limiting conditions and auxotrophy to several amino acids^17,22^.

Despite the presence of (p)ppGpp in majority of free living bacteria, the mechanism of both synthesis and action of this alarmone can differ between bacterial species. In *E*. *coli* the (p)ppGpp-mediated regulation takes place mainly at the level of gene expression, with RNA polymerase as a main target of the alarmone direct and indirect effects. On quite a contrary, in many Gram(+) bacteria, including model *Bacillus subtilis*, (p)ppGpp acts by different strategy; it does not interact directly with RNA polymerase, but its main effect is exerted by controlling the cellular level of GTP^23^. Depleting the cells of GTP, achieved both by lowering the pool of available GTP through (p)ppGpp synthesis and inhibiting the enzymes from GTP synthesis pathway by (p)ppGpp, leads to decreasing of transcription from GTP-starting promoters (including rRNA promoters)^24,25^. This strategy of (p)ppGpp-mediated regulation has been reported for many Gram(+) bacterial species, including *E*. *faecalis*, *S*. *aureus*, *L*. *monocytogenes* and others^26–28^.

The purpose of this work was to evaluate the activity of aliphatic (SFN) and aromatic (PEITC) ITC towards different Gram(−) and Gram(+) bacteria and to correlate it with ppGpp production. First, we compared response to ITC of model strains of *E*. *coli* and *B*. *subtilis*, both wild type and defective in stringent response induction (ppGpp-null variants). Next, we tested activity of ITC toward bacteria belonging to opportunistic human pathogens (*K*. *pneumoniae*, *S*. *aureus*, *S*. *epidermidis*, *E*. *faecalis*) (Table 1). Majority of these species are among the most antibiotic resistant bacteria known, occurring frequently in hospital secondary infections or causing food poisoning.

**Table 1:** Bacterial strains used in this study.

Strain	Reference or source
*E*. *coli* ATCC 25922	ATCC
*E*. *coli* ppGpp-null *∆relA ∆spoT*	^15^
*E*. *coli* 6424	Clinical isolate
*E*. *coli* ESBL KR1557	Clinical isolate
*E*. *coli* ESBL KR45	Clinical isolate
*K*. *pneumoniae* 1077	Clinical isolate
*K*. *pneumoniae* M2836	Clinical isolate
*K*. *pneumoniae* M2801	Clinical isolate
*B*. *subtilis* 168	^36^
*B subtilis* ppGpp-null *relA*::erm ∆*yjbM ywaC*::spc	^37^
*E*. *faecalis* 773	^38^
*E*. *faecalis* M1868	Clinical isolate
*E*. *faecalis* M2056	Clinical isolate
*S*. *aureus* ATCC 25923	ATCC
*S*. *aureus* 6285/10	Clinical isolate
*S*. *aureus* MRSA RA604	Clinical isolate
*S*. *aureus* MRSA RA532	Clinical isolate
*S*. *epidermidis* ATCC 12228	ATCC

## Results

### Comparison of response of *Escherichia coli* and *Bacillus subtilis* model strains to SFN or PEITC

**Figure 1:**
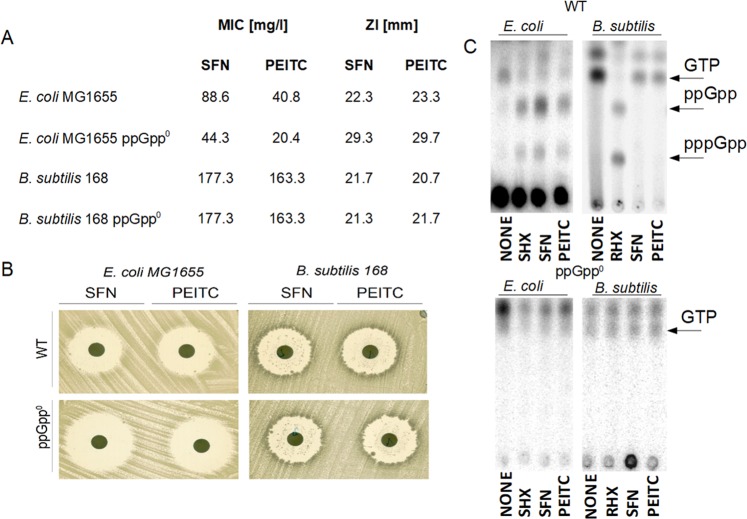
SFN and PEITC effectively inhibit growth of model Gram(−) and Gram(+) bacteria, however it is dependent on stringent response in *E*. *coli* but not in *B*. *subtilis*. Minimal inhibitory concentration (MIC) (**A**) and zone inhibition (ZI) (**A**,**B**) of SFN and PEITC against *E*. *coli* and *B*. *subtilis* strains and their ppGpp^0^ derivatives. For the zone inhibition test discs were spotted aseptically with 0,1 µg of SFN or PEITC. (**C**) The synthesis of ppGpp and pppGpp, and level of GTP were assessed by culturing wt or ppGpp-null bacteria in the presence of [^32^P]orthophosphoric acid followed by cell lysis and nucleotide separation by thin-layer chromatography. SHX or RHX served as positive controls.

It has been shown that some ITCs, including SFN and PEITC, induce the stringent response in *E*. *coli*^15,16^. To determine whether tested ITCs have similar mechanism of action in Gram(+) bacteria, we used *B*. *subtilis* as a model representative of this group. *E*. *coli* cells were more sensitive to tested ITC than *B*. *subtilis*: MIC for SFN was 88.6 and 177.3 mg/L, and for PEITC – 40.8 and 163.3 mg/L, respectively. Interestingly, *E*. *coli* strain unable to produce stringent response alarmones (ppGpp-null) was more sensitive to ITCs than wt strain while sensitivities of wt and ppGpp-null *B*. *subtilis* strains were similar (Fig. 1A). It was confirmed by a zone inhibition assay (Fig. 1A,B). Moreover, neither SFN nor PEITC induced (p)ppGpp accumulation in *B*. *subtilis* which contrasts with the situation observed in *E*. *coli* (Fig. 1C). Interestingly, levels of GTP decreased in both bacteria species when treated with standard stringent control inducer (SHX or RHX) or ITCs, even if (p)ppGpp was not produced in ITC-treated wt strain of *B*. *subtilis* (Fig. 1C).

**Figure 2:**
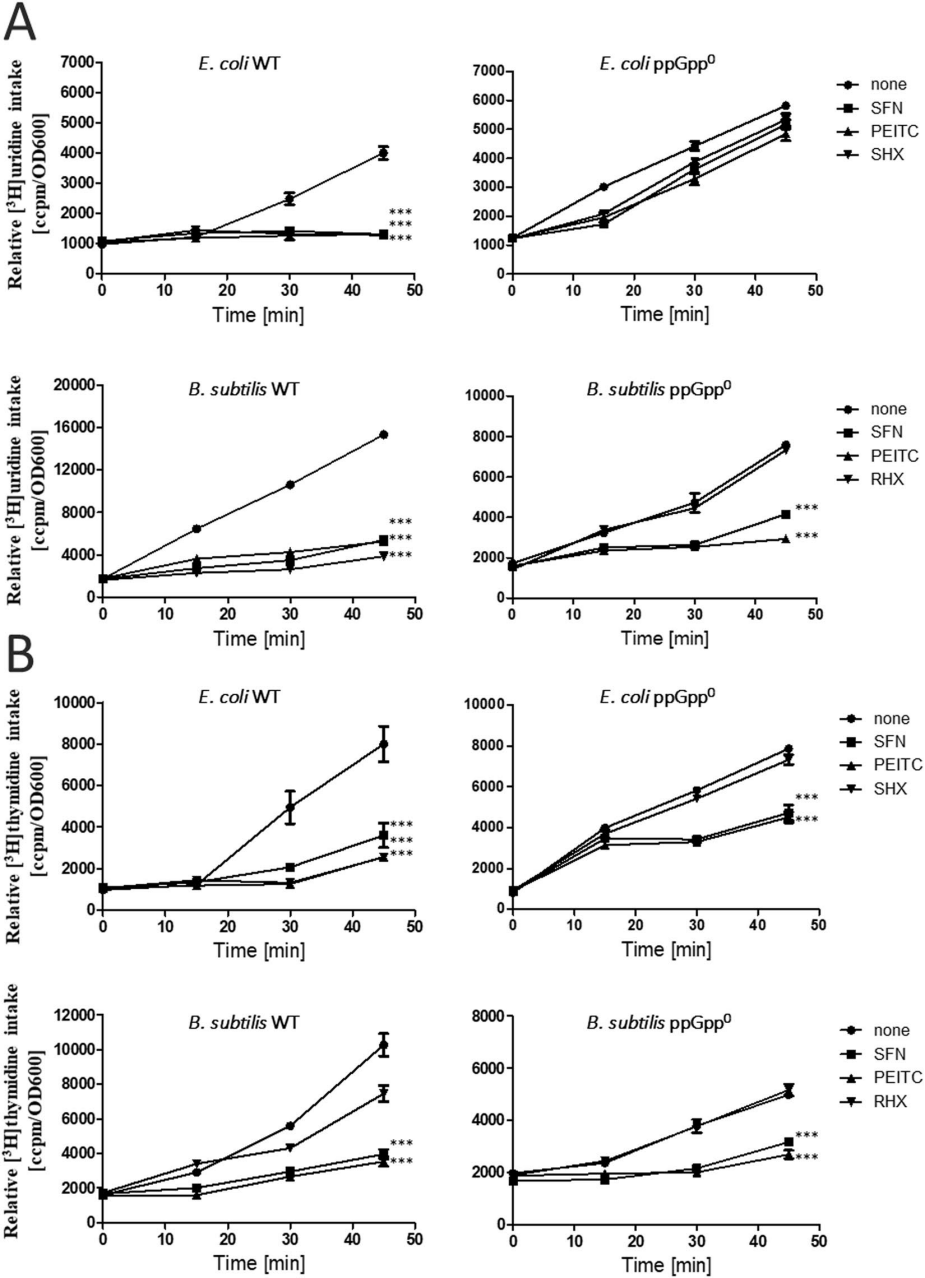
SFN and PEITC modulate synthesis of stable RNA (**A**) and DNA (**B**). *E*. *coli* MG1655 or *B*. *subtilis* 168 (wt) strains and their *relA spoT* (ppGpp^0^) derivatives were grown in a minimal medium at 37 °C, either untreated (none) or in the presence of ITCs, added at time zero to a final concentration of ¼ MIC. [^3^H]uridine (**A**) or [^3^H]thymidine (**B**) was added to 5 μCi, and incorporation into TCA-precipitable material was measured. The results presented are mean values from 3 independent experiments with error bars indicating SD (error bars are not shown when they are smaller than the symbols). The statistical significance of differences between controls and bacteria treated for 120 min with ITC was determined by student t-test, and asterisks indicate significant differences (***P < 0.001).

Induction of the stringent response correlates with the inhibition of synthesis of stable RNA and modulation of DNA synthesis. Thus, we compared nucleic acids synthesis in *E*. *coli* and *B*. *subtilis* and their ppGpp-null counterparts treated or not with ITC at ¼ MIC. In both wild type bacteria strains we observed inhibition of RNA and DNA synthesis upon treatment with SFN, PEITC, and SHX or RHX (positive controls) (Fig. 2). Inhibition of RNA by tested compounds was abrogated in *E*. *coli* ppGpp-null cells which proves that it depends on the stringent response. However, synthesis of RNA in *B*. *subtilis* was inhibited by ITCs irrespective of the presence of this alarmone (Fig. 2A). ITC inhibited DNA synthesis to a lesser extent and it was delayed in time as compared to the effects observed for RNA, thus lack of ppGpp had less pronounced effect on this process (at least at tested time points) (Fig. 2B). It is worth to note that SHX or RHX-induced block in nucleic acids synthesis was abrogated in ppGpp-null strains, both *E*. *coli* and *B*. *subtilis*, which indicates that the stringent response works properly in the wt cells (Fig. 2).

### Lack of the stringent response induction sensitizes *E*. *coli* but not *B*. *subtilis* to ITC

**Figure 3:**
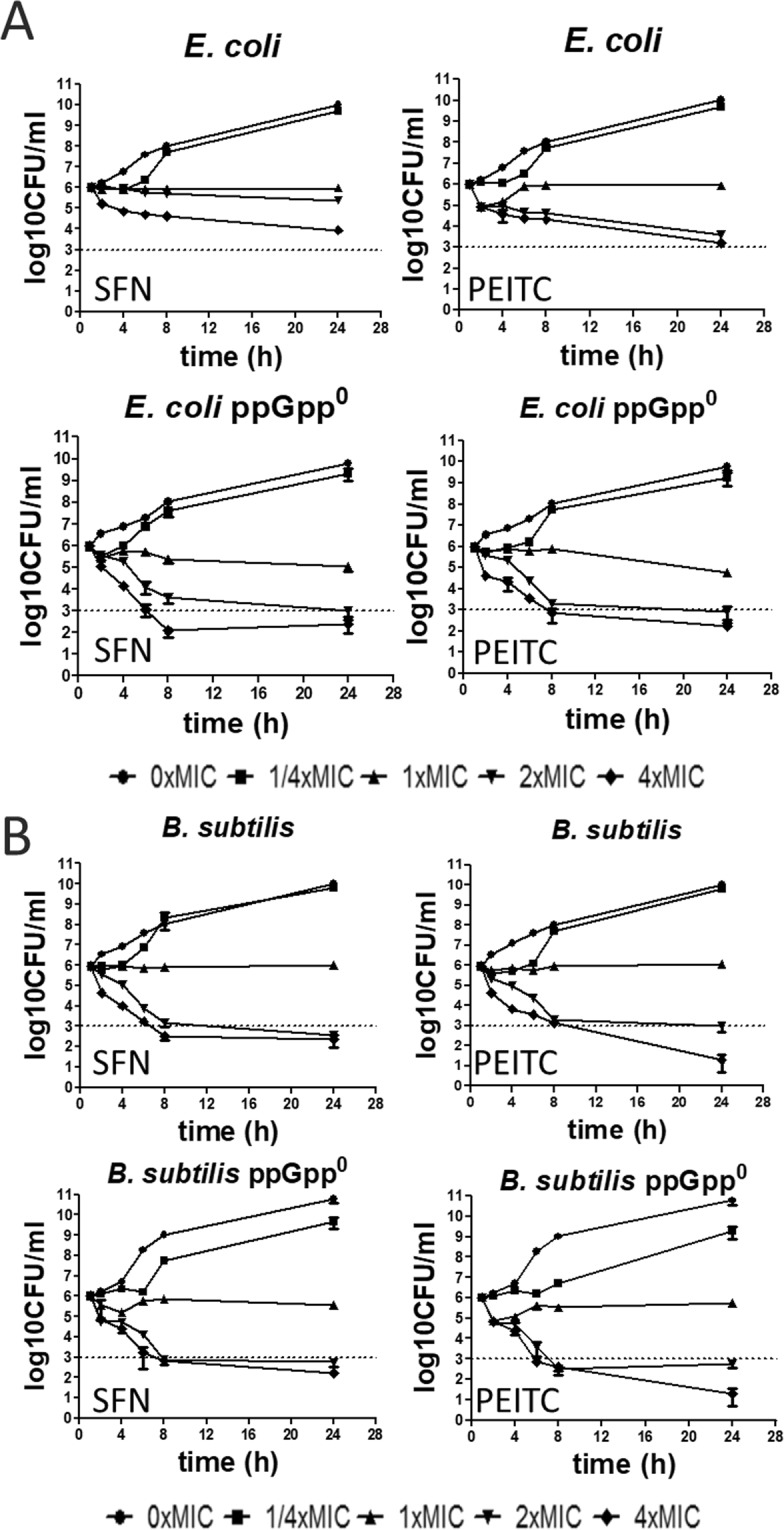
Viability of *E*. *coli* (**A**) and *B*. *subtilis* (**B**) and their ppGpp-null variants is differently affected by SFN or PEITC. Results are shown as the mean ± SD values from 3 experiments. Cultures were treated at 37 °C for 24 h with SFN or PEITC at indicated concentrations. Bactericidal activity was defined as a reduction of 99.9% (≥3 log10) of the total number of CFU mL^-1^ in the original inoculum and marked as dashed line on plots.

As stringent response protects bacteria against adverse conditions, such as shortage of amino acids, we asked the question about its role in bacteria treated with SFN or PEITC. *E*. *coli* and *B*. *subtilis* and their ppGpp-null derivatives were treated with ITC at different concentrations (equivalents of ¼, 1, 2, 4x MIC) and their viability was evaluated at different time points. For *E*. *coli* lack of ppGpp resulted in an increased sensitivity to high concentrations (2 and 4x MIC) of both tested ITCs (Fig. 3A). Sensitivity of *B*. *subtilis* to ITCs did not depend on ppGpp presence which again indicates that response of this bacterium to SFN and PEITC is independent of the stringent response alarmone (Fig. 3B).

### Effect of SFN or PEITC on bacterial growth and stringent response induction in clinical isolates of selected bacteria species

**Figure 4:**
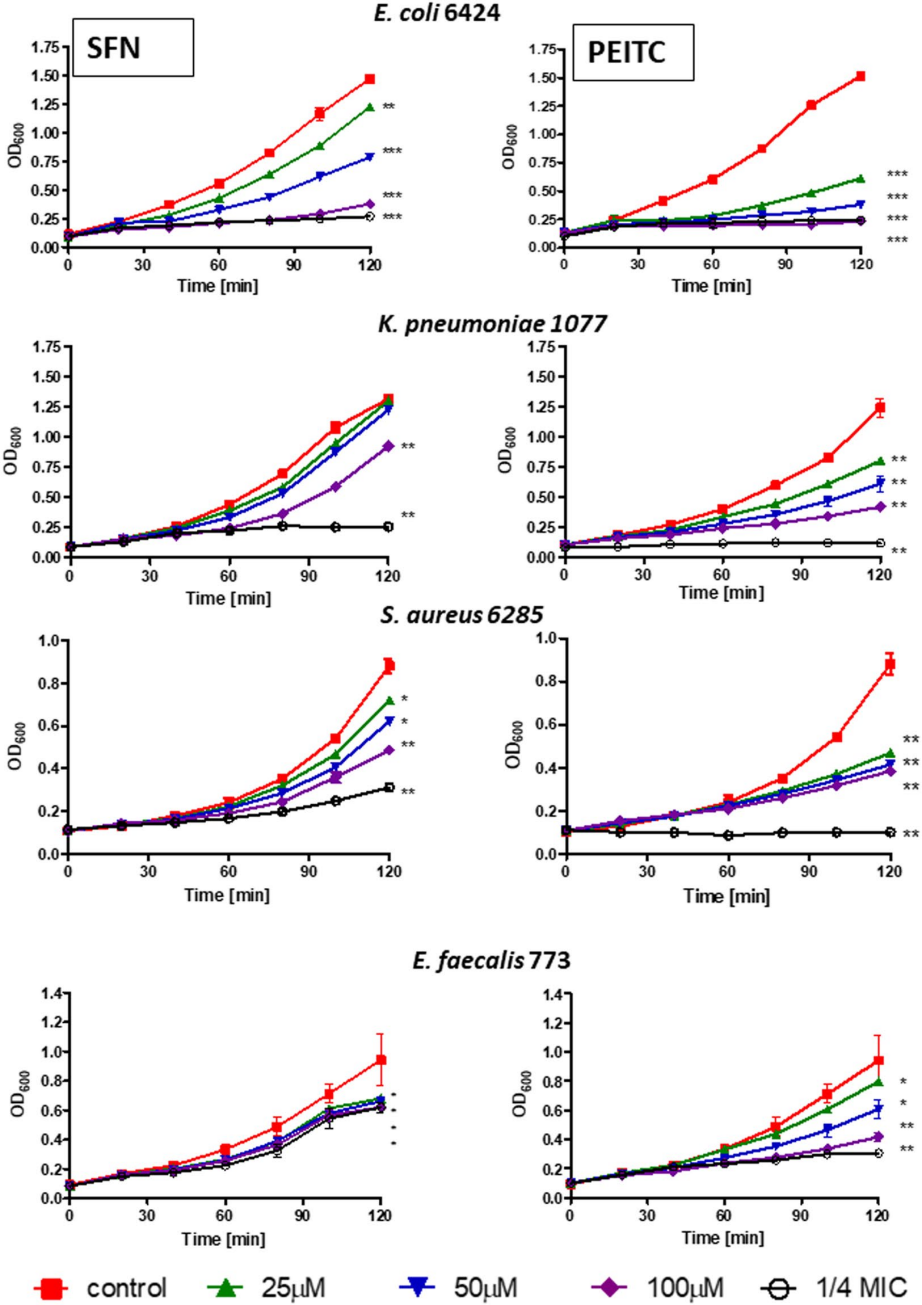
Growth in a rich medium of clinical isolates of different bacteria is inhibited by SFN and PEITC. The results presented are mean values from 3 independent experiments with error bars indicating SD (error bars are not shown when they are smaller than the symbols). The statistical significance of differences between controls and bacteria treated for 120 min with ITC was determined by student t-test, and asterisks indicate significant differences: *P < 0.05; **P < 0.01; ***P < 0.001.

We compared activity of SFN and PEITC on clinical isolates of a broader panel of pathogenic bacteria. Table 2 shows MIC of SFN and PEITC for referenced strains of *E*. *coli*, *S*. *aureus* and *S*. *epidermidis* as well as clinical isolates of *E*. *coli*, *K*. *pneumoniae*, *S*. *aureus* and *E*. *faecalis*. For SFN they are as low as 44 mg/L (*S*. *aureus* reference strain) and as high as 555 mg/L (*K*. *pneumoniae* 1077 clinical isolate) and for PEITC they are as low as 41 mg/L (reference strains of *E*. *coli*, *S*. *aureus* and *S*. *epidermidis*) and as high as 327 mg/L (*K*. *pneumoniae* M2836 and M2801 clinical isolates). Since the composition of growth medium may influence the susceptibility to tested compounds, MIC values were determined for bacteria growing on Mueller-Hinton (MH) or brain heart infusion (BHI) media which are both rich media recommended by the National Committee for Clinical Laboratory Standards. Effect of different concentrations of ITCs on growth kinetics of selected bacteria is shown in Fig. 4. In general, bacterial growth inhibition by ITCs was dose dependent.

**Table 2:** Antimicrobial effect (MIC) of SFN and PEITC against clinical isolates cultivated in Mueller-Hinton (MH) or brain heart infusion (BHI) media.

	SFN [mg/L]		PEITC [mg/L]	
	MH	BHI	MH	BHI
*E*. *coli* ATCC 25922	88.6	88.6	40.8	40.8
*E*. *coli* 6424	254.7	254.7	63.8	63.8
*E*. *coli* ESBL KR1557	177.3	88.6	163.3	163.3
*E*. *coli* ESBL KR45	177.3	177.3	163.3	163.3
*K*. *pneumoniae* 1077	555.0	555.0	254.7	254.7
*K*. *pneumoniae* M2836	177.3	177.3	326.6	326.6
*K*. *pneumoniae* M2801	177.3	177.3	326.6	326.6
*E*. *faecalis* 773	254.7	254.7	127.7	127.7
*E*. *faecalis* M1868	177.3	354.6	81.6	163.3
*E*. *faecalis* M2056	354.6	354.6	163.3	163.3
*S*. *aureus* ATCC 25923	44.3	44.3	40.8	81.6
*S*. *aureus* 6285	88.6	88.6	81.6	81.6
*S*. *aureus* MRSA RA604	177.3	177.3	81.6	81.6
*S*. *aureus* MRSA RA532	177.3	354.6	81.6	163.3
*S*. *epidermidis* ATCC 12228	88.6	177.3	40.8	81.6

**Figure 5:**
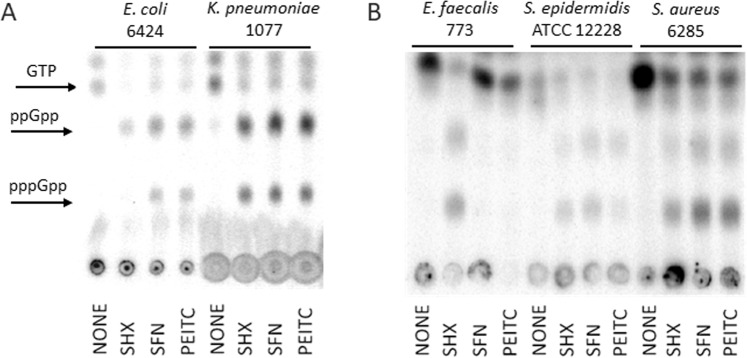
ITCs induce the stringent alarmone synthesis in some but not all tested Gram(−) (**A**) and Gram(+) (**B**) bacteria. The synthesis of ppGpp and pppGpp, and level of GTP were assessed by culturing bacteria in the presence of [^32^P] orthophosphoric acid followed by cell lysis and nucleotide separation by thin-layer chromatography. SHX served as positive control.

**Figure 6:**
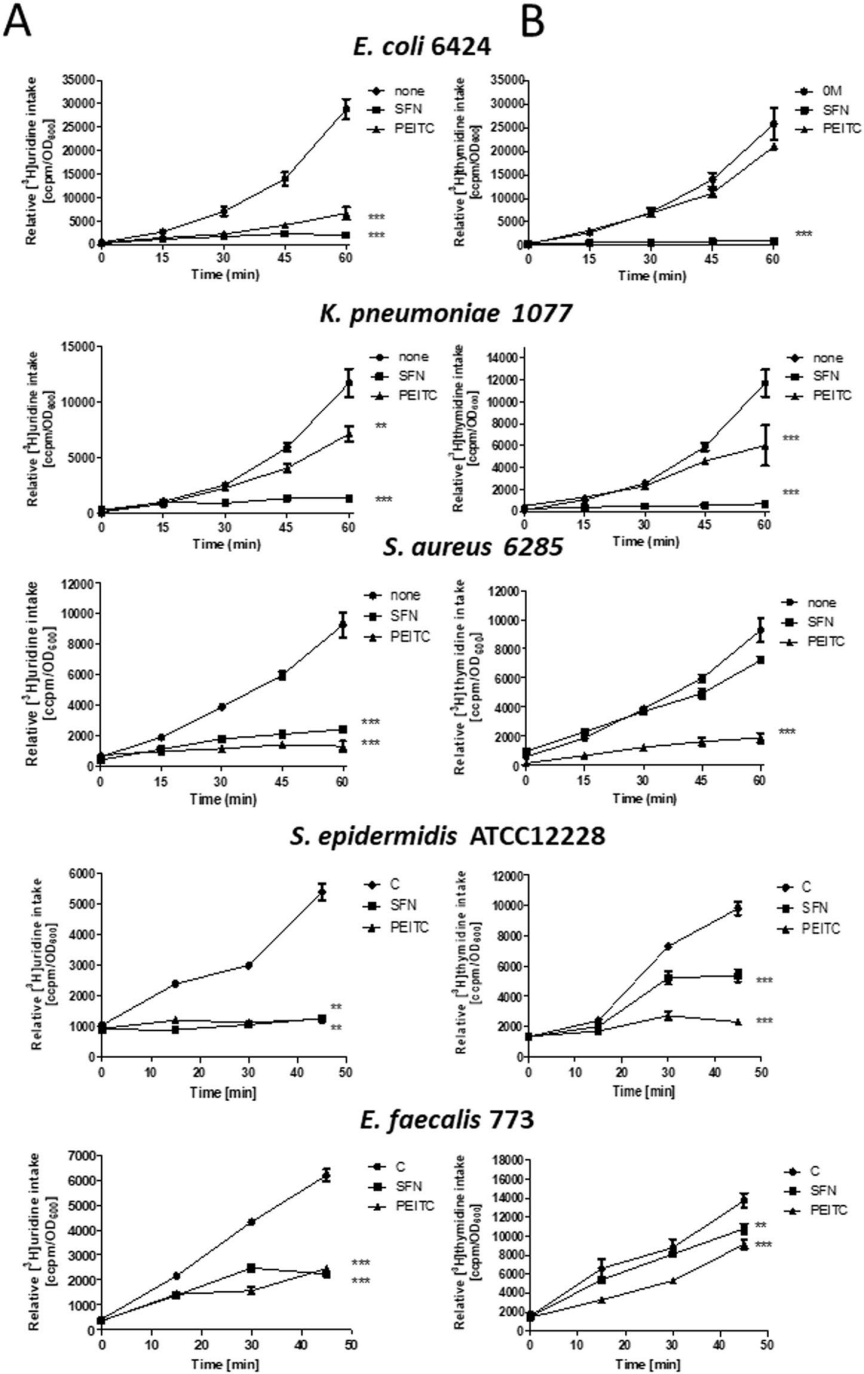
SFN and PEITC modulate synthesis of stable RNA (**A**) and DNA (**B**) in clinical isolates of *E*. *coli* 6424, *K*. *pneumoniae* 1077, *S*. *aureus* 6285 and *E*. *faecalis* 773 as well as *S*. *epidermidis* ATCC12228. Bacteria were grown in a minimal medium at 37 °C, and left untreated (none) or were exposed to ITCs (added at time zero to a final concentration of ¼ MIC). [^3^H]uridine (**A**) or [^3^H]thymidine (**B**) was added to 5 μCi, and incorporation into TCA-precipitable material was measured. The results presented are mean values from 3 independent experiments with error bars indicating SD (error bars are not shown when they are smaller than the symbols). The statistical significance of differences between controls and bacteria treated for 50– 60  min with ITC was determined by student t-test, and asterisks indicate significant differences: **P < 0.01; ***P < 0.001.

To determine whether antibacterial activity of tested ITCs correlates with the stringent response induction in tested bacteria, the levels of (p)ppGpp have been evaluated for selected strains. Results presented in Fig. 5 indicate that SFN and PEITC elevated ppGpp and pppGpp in *E*. *coli*, *K*. *pneumonia*, *S*. *aureus and S*. *epidermidis*. We did not notice the presence of these alarmones in SFN- or PEITC-treated *E*. *faecalis*, although PEITC quite efficiently inhibited growth of this bacterium. SHX, which is a positive control for the stringent response induction, elevated (p)ppGpp in each tested bacterial strain which indicates that stringent response in these isolates functions properly. Interestingly, SFN and PEITC inhibited stable RNA synthesis in all tested bacteria, even in *E*. *faecalis* (Fig. 6A). DNA synthesis was also affected although to a different extent by individual compounds which is connected to indirect effect of the inhibition of gene expression (Fig. 6B).

### Effect of SFN or PEITC on membrane integrity

**Figure 7:**
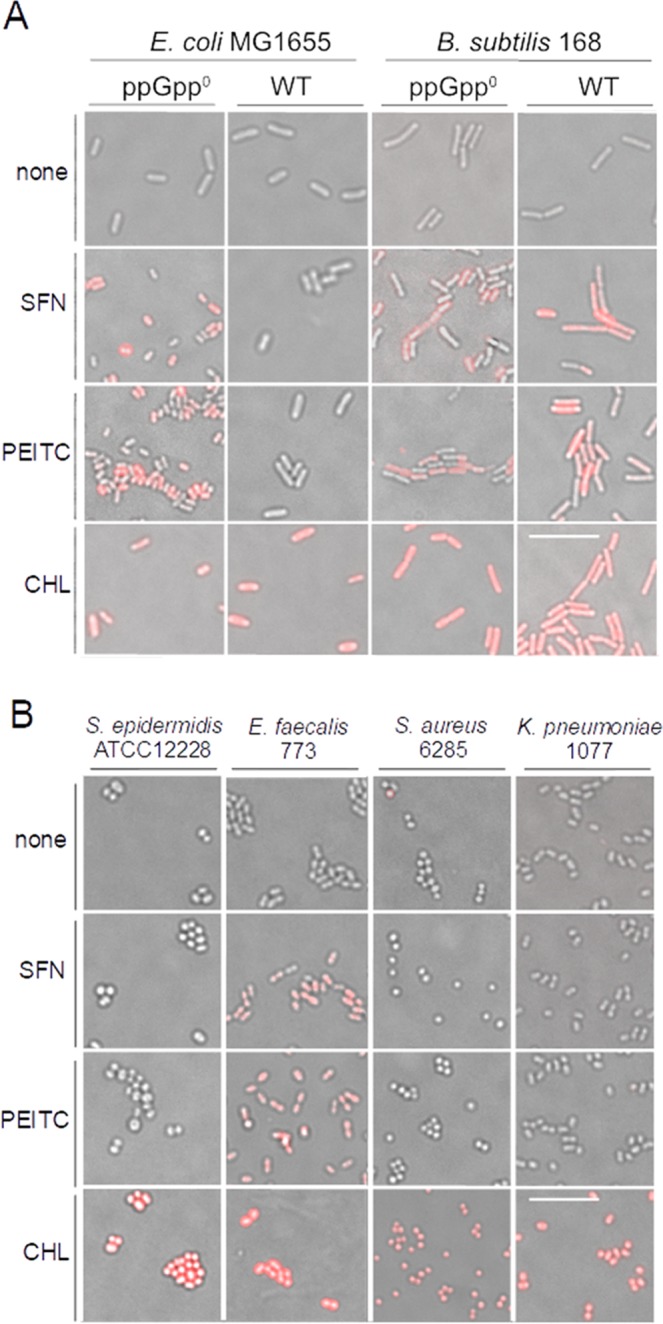
SNF and PEITC affect membrane integrity of some but not all tested bacteria. Fluorescent microscopy analysis of membrane integrity after treatment of (**A**) wt and ppGpp^0^ *E*. *coli* and *B*. *subtilis* strains or (**B**) *S*. *epidermidis* and clinical isolates of selected bacteria. Cells were incubated for 1 h with 4xMIC of ITC or 0.5% chloroform (CHL) and stained with PI; red-fluorescent bacteria have a permeabilized membrane. 10 μm scale bar is the same for all images.

It has been previously suggested that ITC may disturb membrane integrity^13^. Our own results indicated that ITCs, including SFN and PEITC, had no significant effect on membrane integrity in *E*. *coli*^15,16^. However, our experiments were performed in wt strain of *E*. *coli* which was able to induce the stringent response upon ITCs treatment, and the induction of the stringent response may, as an indirect effect, lead to the changes in membrane properties^29^. Thus, we assessed the membrane integrity using propidium iodide test in ppGpp-null *E*. *coli* as well as *B*. *subtilis* and selected clinically isolated bacteria. As evidenced in Fig. 7A, contrary to wild type *E*. *coli* which are impermeable to propidium iodide, the ppGpp-null derivatives stain positively when treated with SFN or PEITC. Interestingly, in *B*. *subtilis* the fluorescence signal indicating the disruption of membrane integrity is visible in both, wt and ppGpp-null strains treated with ITCs. It again supports the notion that ITCs do not induce the stringent response in *B*. *subtilis*, which would otherwise protect against membrane stress. Similarly, *E*. *faecalis* sensitivity to ITCs relies on the loss of membrane integrity probably due to lack of stringent response induction (Fig. 7B). Results presented in Fig. 3S confirm this notion: induction of ppGpp production by RHX protects against membrane permeability induced by ITC in wt *B*. *subtilis*.

## Discussion

Recently we reported that the antibacterial activity of ITCs against enterohaemorrhagic *E*. *coli* relies on the stringent response induction which is connected with massive production of (p)ppGpp, alarmones responsible for, among others, growth retardation and inhibition of stable RNA synthesis or Shiga toxin-converting phage development^15,16^. Here we show that SFN and PEITC inhibit growth of other bacteria, including clinical isolates of *E*. *coli*, *K*. *pneumoniae*, *S*. *aureus*, *S*. *epidermidis*, *E*. *faecalis*. Their sensitivity to ITC varies and the more susceptible are reference than clinically isolated strains: MIC values for *E*. *coli*, *S*. *aureus* or *S*. *epidermidis* ATCC strains are in 44–177 mg/L range for SFN and 41–82 mg/L for PEITC depending on medium, while for their clinical isolates are 82–89 mg/L for *S*. *aureus* 6285 or higher than 555 mg/L for *K*. *pneumoniae* 1077 treated with SFN. Similar MIC values have been reported by others^14^, although Ko *et al*. showed that *E*. *faecalis* is quite resistant to SFN and PEITC (MIC = 1 g/L or higher)^30^. Such discrepancy between MIC values might result from different strains and media used in our study compared with previously published work.

What is interesting, growth inhibition by ITC is independent of (p)ppGpp production in some of tested bacteria. The example is *B*. *subtilis* which does not elevate (p)ppGpp levels after ITC treatment. Interestingly, ITCs in both wild type and ppGpp-null strains of *B*. *subtilis* inhibit nucleic acids synthesis and cell viability. The explanation for this phenomenon might be a drop in GTP level which we observed in ITCs-treated *B*. *subtilis* cells. The regulation of the GTP levels underlies the effects of the stress response of Gram(+) bacteria, e.g. rRNA promoter transcription. The decrease in GTP level under ITC treatment may be responsible for observed RNA synthesis down-regulation. In our case we did not observe (p)ppGpp production in *B*. *subtilis* treated with ITCs, thus it seems that GTP drop is mediated by some other mechanisms. Similar effect, i.e. lack of stringent response induction but inhibition of growth and nucleic acids synthesis by ITCs accompanied by decrease in GTP level, we observed in another Gram(+) bacterium, namely *E*. *faecalis*.

It has been shown that AITC and PEITC were effective against *E*. *coli*, *L*. *monocytogenes*, *S*. *aureus* and *P*. *aeruginosa*, and when used at high concentrations (100, 500 or 1,000 mg/L), they modified physicochemical properties of bacterial surface which led to cytoplasmic membrane permeabilization^14^. Here we confirm that SFN and PEITC compromise the integrity of cytoplasmic membrane, however this is evident only in these strains which do not produce (p)ppGpp upon ITCs treatment, i.e. *B*. *subtilis*, *E*. *faecalis* and *E*. *coli* ppGpp-null variant, and might be the main mechanism underlying antimicrobial activity of ITCs. Interestingly, strains able to induce stringent response, in addition to the observed inhibition of RNA and DNA synthesis, do not react to ITCs treatment with increased membrane permeability, even treated with very high concentrations of ITC (for instance, *K*. *pneumoniae* treated with >2 g/L SFN or 1 g/L PEITC). This is in the agreement with reports showing that ppGpp changes the rate of phospholipid synthesis and increases proportion of saturated to unsaturated fatty acids which results in reduction of membrane permeability in amino acid starved *E*. *coli*^31,32^ and the generalized response to the envelope stress, e.g. in *S*. *aureus*^27^, and in *E*. *coli*^33^. Such response provides a survival advantage in challenging conditions with the example presented by our results  where wild type *E*. *coli* exposed to high concentrations of ITCs (2 or 4x MIC) reveals better survival than its ppGpp-null derivative in prolonged tests. The difference in the response to ITCs treatment dependent on the presence of (p)ppGpp indicates that in the wild type cells induction of the stringent response prevents the membrane disruption by ppGpp-mediated gene expression changes leading to the higher tolerance to the envelope stress.

The question arises about the mechanism of species-specific induction of the stringent response by ITCs. Our previous results indicate that supplementation with high concentrations of specific amino acids reversed inhibitory potential of ITCs. For example, supplementation of glycine, arginine, or to lesser extent lysine, methionine, phenylalanine, serine or threonine restored growth of *E*. *coli* in the presence of PEITC^15^; and glycine, cysteine, arginine, tryptophan and aspartic acid decreased sensitivity to SFN^16^. These results suggest that ITCs might bind and titrate these amino acids or block the aminoacylation of relevant tRNA (e.g. by inhibiting a specific aminoacyltransferase), thus elevating their level in cells would prevent the stringent response induction. It is also possible that in some bacteria titration of specific amino acids by ITCs is not a rate limiting step for stringent response induction, however, such hypothesis needs further experimental validation.

Concluding, our results indicate that ITCs may have more than one target in the bacterial cells, and the mechanism of their antibacterial activity is species-specific. Noteworthy, SFN was reported to have various targets in eukaryotic cells^34^ and modulate numerous cell signalling pathways, some – specific for particular cell line^2^. Here we show that ITCs act in at least two ways in bacteria, but – interestingly – the outcome is similar, namely bacterial growth inhibition. This is of great importance for the potential use of ITCs as the antimicrobial agents in the pathogenic bacteria infections.

## Methods

### Bacterial strains and growth conditions

Bacterial strains used in this study are listed in Table 1. All of clinical strains included in this study were isolated from 2009 until 2017 in Voivodship Hospital in Gdańsk. Bacteria were grown in LB, BHI or MH liquid medium in 37 °C with aeration by shaking or plated on solid medium (LB, BHI and MH supplemented with 1.5% bacteriological agar) and incubated overnight in 37 °C. Serine starvation was induced by addition of serine hydroxamate (SHX) or arginine hydroxamate (RHX) to final concentration of 0.5 mg/mL.

### Susceptibility testing

Antimicrobial activity of SFN and PEITC (LKT Labs, USA) was assessed by determination of the MIC in accordance with CLSI guideline M07-A9. MICs were assessed in two-fold broth microdilution assay and concentrations range from 12.5 to 800 mg/L were used. Zone inhibition tests were assessed by Kirby Bauer disc diffusion method using 6 mm membrane discs (Biomaxima, Poland). Cell concentration of tested microorganisms was adjusted at 0.5 McFarland turbidity standards and inoculated on MHA plates and diameters of growth inhibition were measured after 20-h incubation in 37 °C.

### Evaluation of DNA and RNA synthesis

The assessment of nucleic acids synthesis was performed as described^15^. Briefly, bacteria were grown in MH in 37 °C to A_600_ of 0.1 and the radioactive precursor of DNA or RNA synthesis, [^3^H]thymidine and [^3^H]uridine, respectively, was added at 5 µCi. This time was set as time zero and SFN and PEITC in range of concentrations were added to the cultures. Samples (50 µl) in indicated time were collected on Whatmann 3MM filter papers and transferred to ice-cold 10% trichloroacetic acid (TCA) for 10 min, washed in 5% TCA and in 96% ethanol. After drying of filters, the radioactivity was measured in a scintillation counter MicroBeta (PerkinElmer, Wallac). Results (expressed in cpm) from three independent experiments were normalized to bacterial culture density (A_600_) and presented as mean values with SD.

### Determination of bacterial growth inhibition

Growth inhibition was measured spectrophotometrically (A_600_) in the presence of relevant concentrations of ITCs. Bacterial cultures were grown in 37 °C to early exponential phase A_600_ of 0.1 and treated with indicated compounds. Independent experiments were assessed at least in triplicate and results were expressed as mean ± standard deviation (SD).

### Assessment of (p)ppGpp cellular levels

pppGpp and ppGpp levels were measured basically as described^35^ with modifications as in^15^. Briefly, overnight bacterial plate culture was resuspended in MOPS low phosphate (0.4 mM) minimal medium with the addition of [^32^P]orthophosphoric acid (150 μCi/ml) and grown for at least two generations in 37 °C. Then, culture was divided into new flasks and ITCs (1/4xMIC) or SHX, RHX (1.5 mg/ml both) were added at time zero. The untreated culture was used as a negative control. Bacterial samples, collected at time points, were lysed with formic acid (13 M) in three cycles of freeze-thaw procedure. After centrifugation, nucleotides extracts were separated by thin-layer chromatography, using PEI cellulose plates in 1.5 M potassium phosphate buffer. The chromatograms were analysed in phosphorimager (Typhoon, GE Healthcare).

### Membrane integrity assay

Bacteria were grown with shaking in 37 °C to early exponential phase A_600_ of 0.1 in the same conditions as for growth inhibition experiment. Then, 1 ml of cultures were transferred to Eppendorf tubes 1.5 ml and treated with ITCs in 4xMIC concentration by 1 hour in 37 °C. To determine membrane integrity bacterial cells were washed and resuspended in 1 ml PBS and stained with propidium iodide (IP) 10 µM for 10 min. Bacteria were collected by centrifugation (4,000 × g for 5 min) and washed twice in PBS to remove remaining dye from supernatant. Fluorescence signal from permeabilized cells was analysed using Leica DMI4000B microscope with dedicated filter No. N2.1. Images were collected and processed using LAS AF 3.1 software (Leica). Experiments were repeated at least three times. Results were expressed on photographs as merge of transmitted light- Differential Interference Contrast (DIC) and fluorescence channel.

## Supplementary information

Supplementary data

## Data Availability

All data generated or analyzed during this study are included in this published article and Supplementary Information File.
