# Supplementary material for: Various modes of action of dietary phytochemicals, sulforaphane and phenethyl isothiocyanate, on pathogenic bacteria
Source: Sci Rep. 2019 Sep 23;9:13677. doi: 10.1038/s41598-019-50216-x (PMC6757042; doi:10.1038/s41598-019-50216-x)

Various modes of action of dietary phytochemicals, sulforaphane and phenethyl isothiocyanate, on pathogenic bacteria

Nowicki D., Maciag-Dorszynska M., Bogucka K., Szalewska-Pałasz A., Herman-Antosiewicz A.

Figure 1S. Induction of the stringent response by increasing amounts of ITCs in *B. subtilis* 168 WT and $ppGpp^0$ strain. The synthesis of the stringent response alarmones, ppGpp and pppGpp was assessed by culturing bacteria in the presence of [32P]orthophosphoric acid (150 µCi/ml) followed by cell lysis and nucleotide separation by thin-layer chromatography. ITCs (as indicated above each panel) or RHX (lane 2) or SHX (lane 3) were added at time zero and samples were withdrawn at 30' and 60' after the addition. ITCs were added the final concentrations of 4 x MIC (lanes 4,7), 1 x MIC (lanes 5,8), 1/2 (lanes 6,9) 1/8 MIC (lane 10). The positions of ppGpp, pppGpp, GTP, GDP and ATP are indicated by arrows

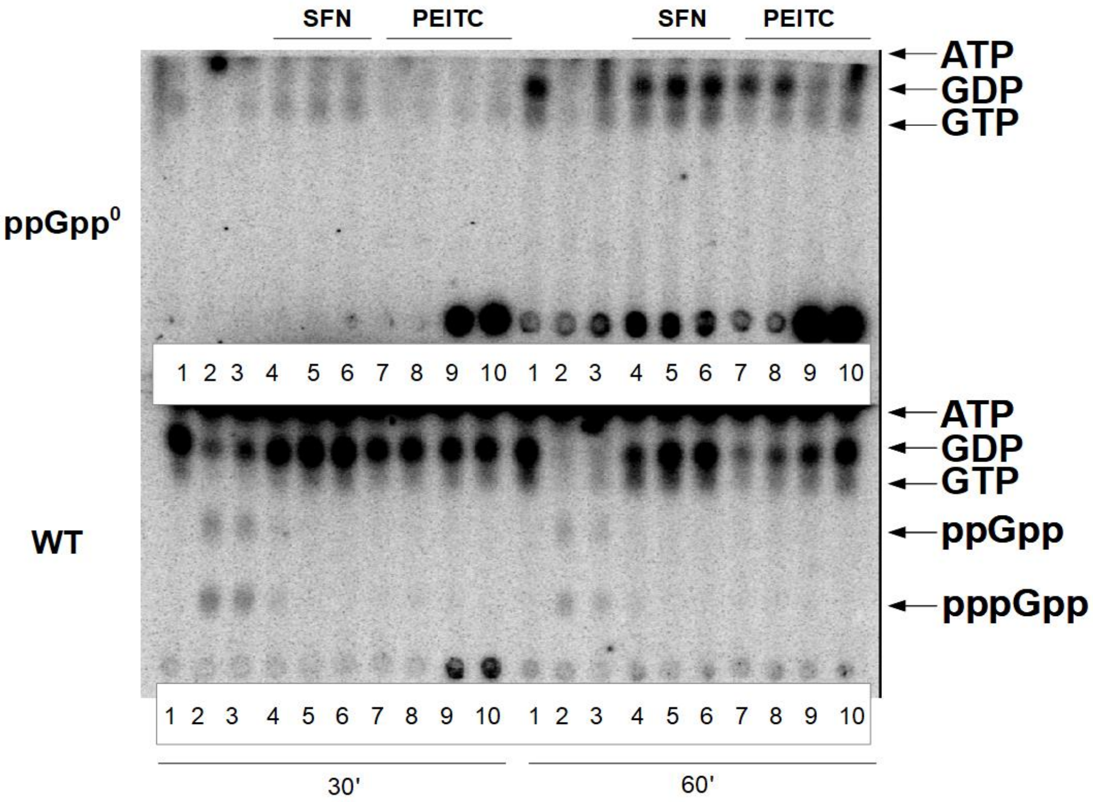

Figure 2S. Induction of the stringent response by SFN and PEITC. The full length of chromatograms presented in the original manuscript on Figure 1 and Figure 5.

1- untreated culture
2- SHX
3- SFN
4- PEITC
5- RHX

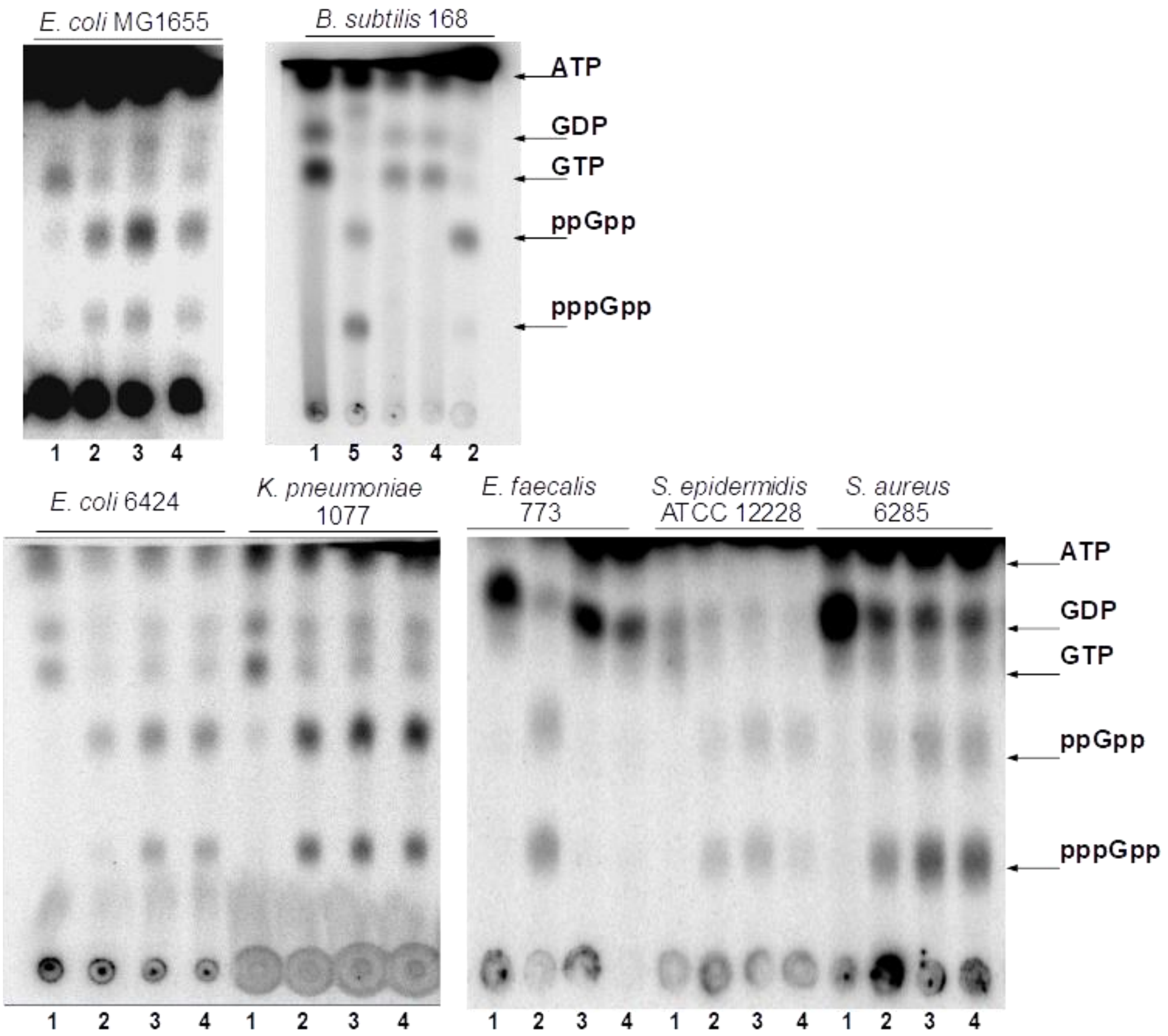

Figure 3S. The impact of ITCs on membrane disruption in starved bacterial *E. coli* and *B. subtilis* cells. Fluorescent microscopy analysis of membrane integrity after SNF and PEITC treatment of WT and $ppgpp^0$ E. coli and B. subtilis strains. Cells were incubated 1h with 4xMIC of ITC and 0.5% chlorophorm (CM) and stained with PI; To mimic the amino acid starvation conditions SHX or RHX (0,5mg/ml) was added to the cultures in t=0. Red-fluorescent bacteria have a permeabilized membrane indicated by arrows. Treated cells were washed four times with high-salt solution, and 3 µl was placed on 1% agarose slice between two cover glasses to obtain an unmovable monolayer of cells. 10 µm scale bar is the same for all images.

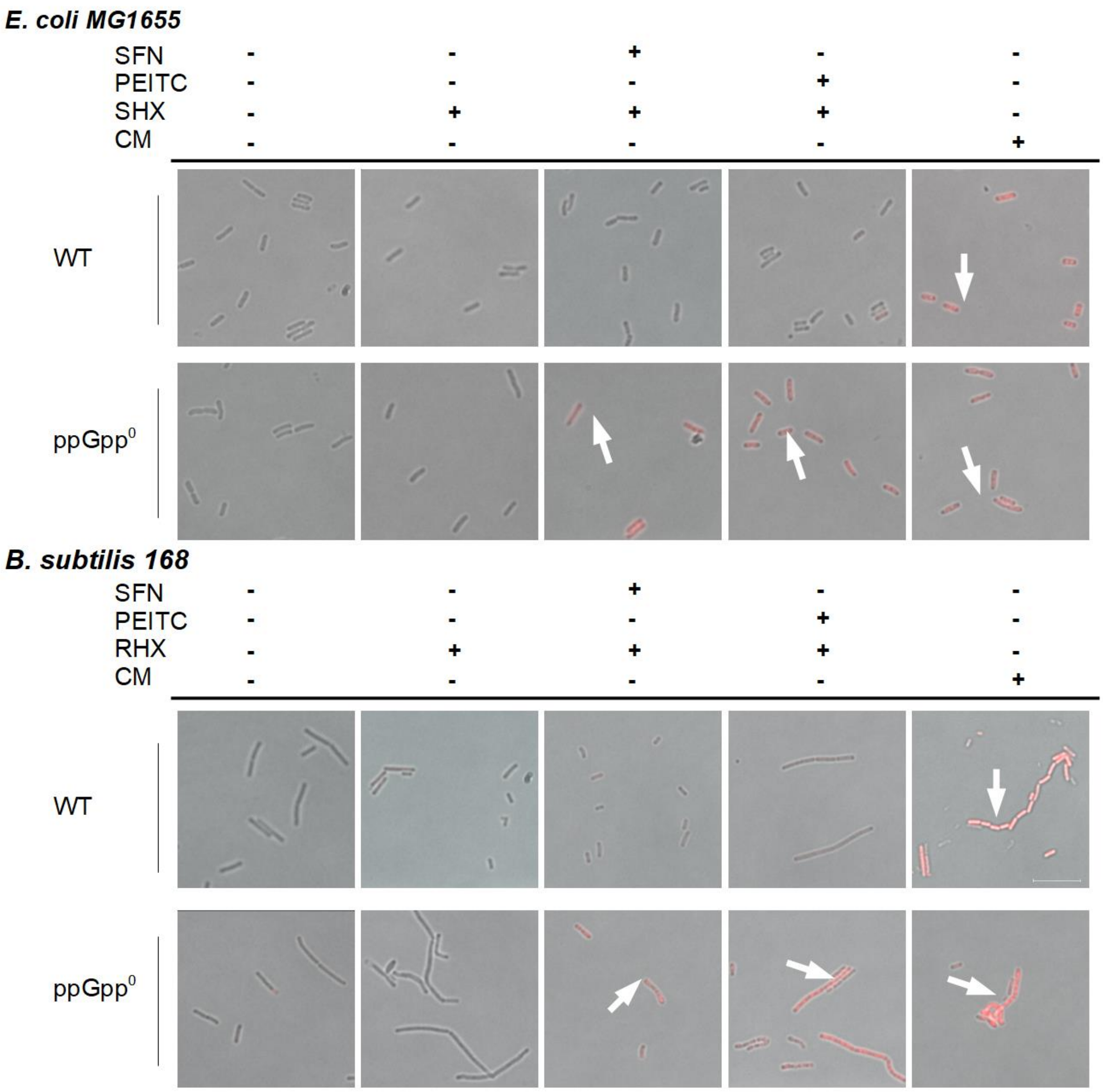

Supplement: Supplementary file 1 — Supplementary data [file 41598_2019_50216_MOESM1_ESM.pdf]
